# 2-Fluoro-4-(meth­oxy­carbon­yl)benzoic acid

**DOI:** 10.1107/S1600536810032253

**Published:** 2010-08-18

**Authors:** Carl E. Wagner, Thomas L. Groy

**Affiliations:** a Division of Mathematics and Natural Sciences, Arizona State University, West Campus, Glendale, AZ 85306, USA; b Department of Chemistry and Biochemistry, Arizona State University, Tempe, AZ 85287-1604, USA

## Abstract

In the crystal of the title compound, C_9_H_7_FO_4_, classical carboxylate inversion dimers are linked by pairs of O—H⋯O hydrogen bonds.  The packing is consolidated by C—H⋯F and C—H⋯O interactions. The benzene ring and the methoxycarbonyl group are nearly coplanar, with a dihedral angle of 1.5 (3)° between them, whereas the carboxyl group has a dihedral angle of 20.2 (4)° with respect to the benzene ring.

## Related literature

For background to the applications of the title compound, see: Jiang *et al.* (2008 ▶); Sakaki *et al.* (2007 ▶). For related structures, see: Wagner *et al.* (2009 ▶).
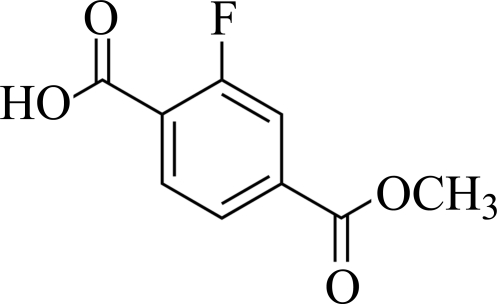


## Experimental

### 

#### Crystal data

C_9_H_7_FO_4_
*M* *_r_* = 198.15
Triclinic, 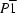

*a* = 7.536 (7) Å
*b* = 7.591 (7) Å
*c* = 8.523 (8) Å
α = 99.480 (14)°
β = 108.748 (13)°
γ = 99.240 (14)°
*V* = 443.3 (7) Å^3^
*Z* = 2
Mo *K*α radiation
μ = 0.13 mm^−1^
*T* = 296 K
0.25 × 0.19 × 0.08 mm

#### Data collection

Bruker SMART APEX CCD diffractometer
Absorption correction: multi-scan (*SADABS*; Bruker, 2008 ▶) *T*_min_ = 0.969, *T*_max_ = 0.990
2526 measured reflections
1535 independent reflections
1025 reflections with *I* > 2σ(*I*)
*R*_int_ = 0.025

#### Refinement

*R*[*F* ^2^ > 2σ(*F* ^2^)] = 0.066
*wR*(*F* ^2^) = 0.190
*S* = 1.02
1535 reflections
128 parameters
H-atom parameters constrained
Δρ_max_ = 0.24 e Å^−3^
Δρ_min_ = −0.22 e Å^−3^

### 

Data collection: *APEX2* (Bruker, 2008 ▶); cell refinement: *SAINT-Plus* (Bruker, 2008 ▶); data reduction: *SAINT-Plus*; program(s) used to solve structure: *SHELXS97* (Sheldrick, 2008 ▶); program(s) used to refine structure: *SHELXL97* (Sheldrick, 2008 ▶); molecular graphics: *XSHELL* (Bruker, 2004 ▶); software used to prepare material for publication: *APEX2*.

## Supplementary Material

Crystal structure: contains datablocks global, I. DOI: 10.1107/S1600536810032253/pb2036sup1.cif

Structure factors: contains datablocks I. DOI: 10.1107/S1600536810032253/pb2036Isup2.hkl

Additional supplementary materials:  crystallographic information; 3D view; checkCIF report

## Figures and Tables

**Table 1:** Hydrogen-bond geometry (Å, °)

*D*—H⋯*A*	*D*—H	H⋯*A*	*D*⋯*A*	*D*—H⋯*A*
C9—H9*A*⋯F1^i^	0.96	2.54	3.278 (5)	134 (1)
O2^ii^—H2*A*^ii^⋯O1	0.82	1.86	2.672 (4)	170 (1)
C3—H3*A*⋯O3^iii^	0.93	2.53	3.325 (4)	144 (1)

Symmetry codes: (i) 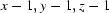; (ii) 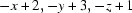; (iii) 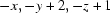.

**Fig. 1.**
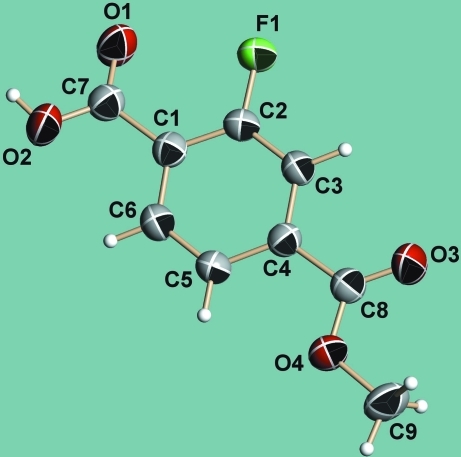
Labeled thermal ellipsoid plot of (1) shown at the 50% probability level for all non-H atoms.

**Fig. 2.**
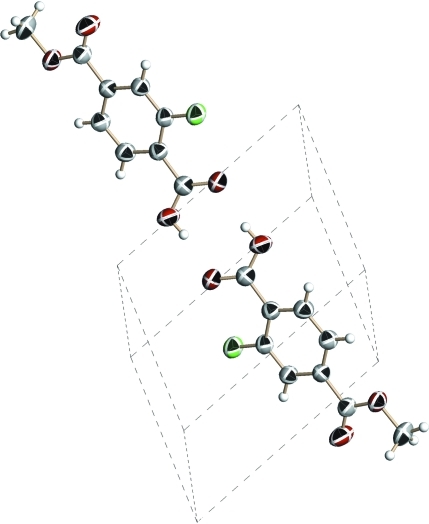
Molecular pair of (1) shown at the 50% probability level for all non-H atoms illustrating classical intermolecular centrosymmetric carboxylic acid hydrogen bonding interactions.

**Fig. 3.**
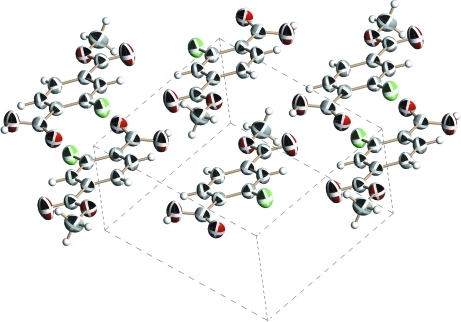
Packing diagram of (1) shown at the 50% probability level for all non-H atoms showing the alternating molecular orientations in two adjacent layers.

## supplementary crystallographic information

### Comment

The title compound, 4-(methoxycarbonyl)-2-fluorobenzoic acid, has recently been used to prepare novel diazepinylbenzoic acid retinoid-X-receptor antagonists (Jiang *et al.*, 2008; Sakaki *et al.*, 2007) as potential oral anti-obesity and anti-diabetic treatments as well as novel retinoid-X-receptor agonists with potential to treat various human cancers. Thus, the X-ray diffraction data of the present study confirms the fluorine locus for 4-(methoxycarbonyl)-2-fluorobenzoic acid.

The structure consists of sheets parallel to (212) stabilized by six intermolecular hydrogen interactions per molecule as shown in Table 1. The benzene ring and the methoxycarbonyl group are essentially coplanar as shown by the 1.51 (25)° dihedral angle between the two planes. However, the carboxylic acid is not coplanar with the benzene ring, as shown by the 20.18 (36)° dihedral angle between those two planes.

### Experimental

The method of Sakaki and co-workers (Sakaki *et al.*, 2007) was followed to synthesize (1). To a flask containing 3-fluoro-4-formylmethylbenzoate (Wagner *et al.*, 2009) (9.22 g, 50.5 mmol) and sulfamic acid (5.40 g, 55.6 mmol) in water (21 ml) and ACN (42 ml) was slowly added a solution of 80% NaClO_2_ (4.92 g, 53.8 mmol) in water (21 ml) at room temperature. After being stirred for 1 h, the reaction solution was added to a saturated, aqueous solution of Na_2_SO_3_ (75 ml) and 1 N HCl (150 ml), and the resulting solution was extracted with ethyl acetate (75 ml) three times. The combined organic extracts were washed with brine, dried over sodium sulfate, and the solvents were removed *in vacuo* to give crude (1) (7.56 g, 75%) as a white solid. A small sample was crystallized from hot ethyl acetate to give pure (1) as white crystals, m.p. 154–155 °C: ^1^H NMR (400 MHz, CDCl_3_) δ 10.5 (br s, 1H), 8.10 (t, *J* = 7.8, 1H), 7.89 (d, *J* = 8.2, 1H), 7.82 (d, *J* = 11.0, 1H), 3.97 (s, 3H); ^13^C NMR (100.6 MHz, CDCl_3_) δ 168.6, 168.5, 165.0, 164.9, 163.4, 160.8, 136.7, 136.6, 132.8, 124.9, 124.8, 121.3, 121.2, 118,4, 118.1, 52.8; LC-APCI-MS (*M*+) calcd for C_9_H_7_O_4_F 198.0328, found 198.0331.

### Refinement

H atoms were placed geometrically and allowed to refine as atoms riding on their bonding partners. The hydrogen was placed on the carboxylic acid based on the longer of the carboxylic acid carbon-oxygen bonds.

### Crystal data

**Table:**

C_9_H_7_FO_4_	*Z* = 2
*M**_r_* = 198.15	*F*(000) = 204
Triclinic, *P*1	*D*_x_ = 1.484 Mg m^−^^3^
Hall symbol: -P 1	Melting point: 427 K
*a* = 7.536 (7) Å	Mo *K*α radiation, λ = 0.71073 Å
*b* = 7.591 (7) Å	Cell parameters from 51 reflections
*c* = 8.523 (8) Å	θ = 4.5–11.9°
α = 99.480 (14)°	µ = 0.13 mm^−^^1^
β = 108.748 (13)°	*T* = 296 K
γ = 99.240 (14)°	Plate, colourless
*V* = 443.3 (7) Å^3^	0.25 × 0.19 × 0.08 mm

### Data collection

**Table:**

Bruker SMART APEX CCD diffractometer	1535 independent reflections
Radiation source: sealed tube	1025 reflections with *I* > 2σ(*I*)
graphite	*R*_int_ = 0.025
ω and φ scans	θ_max_ = 25.0°, θ_min_ = 2.6°
Absorption correction: multi-scan (*SADABS*; Bruker, 2008)	*h* = −8→8
*T*_min_ = 0.969, *T*_max_ = 0.990	*k* = −9→8
2526 measured reflections	*l* = −10→10

### Refinement

**Table:**

Refinement on *F*^2^	Primary atom site location: structure-invariant direct methods
Least-squares matrix: full	Secondary atom site location: difference Fourier map
*R*[*F*^2^ > 2σ(*F*^2^)] = 0.066	Hydrogen site location: inferred from neighbouring sites
*wR*(*F*^2^) = 0.190	H-atom parameters constrained
*S* = 1.02	*w* = 1/[σ^2^(*F*_o_^2^) + (0.*P*)^2^ + 0.1145*P*] where *P* = (*F*_o_^2^ + 2*F*_c_^2^)/3
1535 reflections	(Δ/σ)_max_ = 0.001
128 parameters	Δρ_max_ = 0.24 e Å^−^^3^
0 restraints	Δρ_min_ = −0.22 e Å^−^^3^

### Special details

**Table:**

Geometry. All e.s.d.'s (except the e.s.d. in the dihedral angle between two l.s. planes) are estimated using the full covariance matrix. The cell e.s.d.'s are taken into account individually in the estimation of e.s.d.'s in distances, angles and torsion angles; correlations between e.s.d.'s in cell parameters are only used when they are defined by crystal symmetry. An approximate (isotropic) treatment of cell e.s.d.'s is used for estimating e.s.d.'s involving l.s. planes.
Refinement. Refinement of *F*^2^ against ALL reflections. The weighted *R*-factor *wR* and goodness of fit *S* are based on *F*^2^, conventional *R*-factors *R* are based on *F*, with *F* set to zero for negative *F*^2^. The threshold expression of *F*^2^ > σ(*F*^2^) is used only for calculating *R*-factors(gt) *etc*. and is not relevant to the choice of reflections for refinement. *R*-factors based on *F*^2^ are statistically about twice as large as those based on *F*, and *R*- factors based on ALL data will be even larger.H atoms were placed geometrically and allowed to refine as atoms riding on their bonding partners. The hydrogen was placed on the carboxylic acid based on the longer of the carboxylic acid carbon-oxygen bonds.

### Fractional atomic coordinates and isotropic or equivalent isotropic displacement parameters (Å^2^)

**Table:**

	*x*	*y*	*z*	*U*_iso_*/*U*_eq_	
F1	0.4739 (2)	1.3542 (2)	0.6014 (2)	0.0629 (5)	
O1	0.8299 (2)	1.4232 (4)	0.5841 (2)	0.0672 (7)	
O2	0.8296 (2)	1.3413 (2)	0.3192 (2)	0.0716 (8)	
H2A	0.9326	1.4171	0.3603	0.107*	
O3	−0.1230 (4)	0.8633 (4)	0.2593 (2)	0.0798 (9)	
O4	−0.0485 (2)	0.7256 (2)	0.0437 (2)	0.0582 (7)	
C1	0.5568 (4)	1.2070 (4)	0.3734 (2)	0.0453 (7)	
C2	0.4248 (4)	1.2212 (4)	0.4562 (2)	0.0464 (7)	
C3	0.2443 (4)	1.1083 (4)	0.3967 (2)	0.0479 (8)	
H3A	0.1607	1.1227	0.4553	0.057*	
C4	0.1871 (4)	0.9720 (4)	0.2478 (2)	0.0437 (7)	
C5	0.3166 (4)	0.9507 (4)	0.1625 (2)	0.0495 (8)	
H5A	0.2807	0.8584	0.0642	0.059*	
C6	0.4976 (5)	1.0670 (4)	0.2248 (4)	0.0527 (8)	
H6A	0.5819	1.0523	0.1669	0.063*	
C7	0.7527 (4)	1.3333 (4)	0.4321 (4)	0.0503 (8)	
C8	−0.0112 (4)	0.8498 (4)	0.1877 (4)	0.0491 (8)	
C9	−0.2360 (5)	0.5972 (5)	−0.0216 (4)	0.0692 (10)	
H9A	−0.2482	0.5131	−0.1238	0.104*	
H9B	−0.3359	0.6639	−0.0462	0.104*	
H9C	−0.2474	0.5303	0.0623	0.104*	

### Atomic displacement parameters (Å^2^)

**Table:**

	*U*^11^	*U*^22^	*U*^33^	*U*^12^	*U*^13^	*U*^23^
F1	0.0579 (11)	0.0628 (13)	0.0540 (10)	−0.0020 (9)	0.0211 (9)	−0.0111 (8)
O1	0.0526 (15)	0.0782 (17)	0.0532 (13)	−0.0072 (11)	0.0119 (10)	0.0038 (11)
O2	0.0590 (16)	0.0765 (18)	0.0690 (15)	−0.0133 (11)	0.0303 (11)	0.0019 (11)
O3	0.0532 (16)	0.088 (2)	0.0828 (17)	−0.0110 (13)	0.0346 (13)	−0.0154 (14)
O4	0.0485 (14)	0.0591 (14)	0.0525 (11)	−0.0015 (10)	0.0137 (10)	−0.0038 (10)
C1	0.0435 (17)	0.0453 (17)	0.0477 (15)	0.0102 (14)	0.0161 (13)	0.0123 (13)
C2	0.0485 (18)	0.0445 (17)	0.0414 (14)	0.0071 (13)	0.0153 (13)	0.0026 (11)
C3	0.0447 (18)	0.051 (2)	0.0470 (15)	0.0086 (14)	0.0202 (13)	0.0045 (13)
C4	0.0431 (17)	0.0430 (16)	0.0434 (15)	0.0074 (13)	0.0149 (13)	0.0091 (11)
C5	0.050 (2)	0.0467 (18)	0.0490 (16)	0.0076 (14)	0.0208 (14)	0.0017 (13)
C6	0.050 (2)	0.056 (2)	0.0535 (17)	0.0099 (15)	0.0243 (14)	0.0063 (14)
C7	0.049 (2)	0.0493 (18)	0.0510 (17)	0.0095 (14)	0.0168 (15)	0.0105 (14)
C8	0.0432 (18)	0.0511 (18)	0.0490 (16)	0.0079 (14)	0.0156 (14)	0.0055 (13)
C9	0.052 (2)	0.062 (2)	0.069 (2)	−0.0055 (17)	0.0060 (16)	−0.0018 (17)

### Geometric parameters (Å, °)

**Table:**

F1—C2	1.364 (3)	C3—C4	1.393 (4)
O1—C7	1.257 (3)	C3—H3A	0.93
O2—C7	1.278 (4)	C4—C5	1.405 (4)
O2—H2A	0.82	C4—C8	1.504 (4)
O3—C8	1.197 (4)	C5—C6	1.384 (4)
O4—C8	1.336 (4)	C5—H5A	0.93
O4—C9	1.460 (4)	C6—H6A	0.93
C1—C2	1.400 (4)	C9—H9A	0.96
C1—C6	1.405 (4)	C9—H9B	0.96
C1—C7	1.504 (4)	C9—H9C	0.96
C2—C3	1.372 (4)		
			
C7—O2—H2A	109.5	C4—C5—H5A	120.0
C8—O4—C9	115.8 (2)	C5—C6—C1	121.4 (3)
C2—C1—C6	116.8 (3)	C5—C6—H6A	119.3
C2—C1—C7	124.1 (3)	C1—C6—H6A	119.3
C6—C1—C7	119.2 (2)	O1—C7—O2	124.2 (3)
F1—C2—C3	117.5 (2)	O1—C7—C1	119.9 (3)
F1—C2—C1	119.5 (3)	O2—C7—C1	115.9 (3)
C3—C2—C1	122.9 (3)	O3—C8—O4	124.0 (3)
C2—C3—C4	119.5 (3)	O3—C8—C4	124.0 (3)
C2—C3—H3A	120.2	O4—C8—C4	112.0 (2)
C4—C3—H3A	120.2	O4—C9—H9A	109.5
C3—C4—C5	119.4 (3)	O4—C9—H9B	109.5
C3—C4—C8	117.9 (2)	H9A—C9—H9B	109.5
C5—C4—C8	122.7 (3)	O4—C9—H9C	109.5
C6—C5—C4	120.0 (3)	H9A—C9—H9C	109.5
C6—C5—H5A	120.0	H9B—C9—H9C	109.5

### Hydrogen-bond geometry (Å, °)

**Table:**

*D*—H···*A*	*D*—H	H···*A*	*D*···*A*	*D*—H···*A*
C9—H9A···F1^i^	0.96	2.54	3.278 (5)	134 (1)
O2^ii^—H2A^ii^···O1	0.82	1.86	2.672 (4)	170 (1)
C3—H3A···O3^iii^	0.93	2.53	3.325 (4)	144 (1)

Symmetry codes:  (i) *x*−1, *y*−1, *z*−1; (ii) −*x*+2, −*y*+3, −*z*+1; (iii) −*x*, −*y*+2, −*z*+1.
